# Palliative care for adolescents and young adults with cancer

**DOI:** 10.2147/COAYA.S29757

**Published:** 2013-03-24

**Authors:** Abby R Rosenberg, Joanne Wolfe

**Affiliations:** 1 Division of Pediatric Hematology/ Oncology, Seattle Children’s Hospital, Seattle, WA; 2 Clinical Research Division, Fred Hutchinson Cancer Research Center, Seattle, WA; 3 Department of Pediatrics, University of Washington, Seattle, WA; 4 Department of Psychosocial Oncology and Palliative Care/Division of Pediatric Palliative Care, Dana-Farber Cancer Institute, Boston, MA; 5 Department of Medicine/Division of Hematology/Oncology, Boston Children’s Hospital, Boston, MA; 6 Department of Pediatrics, Harvard University School of Medicine, Boston, MA, USA

**Keywords:** supportive care, end of life, psychosocial outcomes, psychosocial oncology, psychosocial needs, quality of life, pediatric oncology

## Abstract

Adolescents and young adults (AYAs) with cancer represent a unique and challenging group of patients with distinct developmental and psychosocial needs that may be unrecognized or unmet during their cancer experience. Palliative care refers to the total care of a patient, regardless of his or her disease status, and aims to improve quality of life by controlling symptoms and alleviating physical, social, psychological, and spiritual suffering. Integrating palliative care into standard oncology practice for AYAs is therefore valuable, if not imperative, in improving their overall cancer experience. In this review, we aimed to describe the scope, benefits, and challenges of palliative care for AYA oncology patients. We provide a broad impression of the existing literature describing or investigating palliative care in this population. Put together, the evidence suggests that palliative care is not only needed, but can also be critically beneficial to patients, families, and health care professionals alike. As we increase public and professional awareness of the needs and applications of palliative care for AYA patients with cancer, we will ultimately enable better psychosocial outcomes of the AYA patients and their larger communities.

## Introduction to palliative care in adolescents and young adults with cancer

Adolescent and young adult (AYA) patients with cancer represent a unique and challenging group. While the incidence of cancer among AYAs has steadily increased over the past 25 years,^1^ these patients have not seen the same improvements in survival as their younger pediatric or older adult counterparts.^2–4^ This so-called AYA gap has been attributed to a variety of factors, including delays in diagnosis,^5^ biological factors,^6^ and poor accrual of AYA oncology patients into clinical trials.^7^ In addition, AYA cancer survivors have inferior psychosocial outcomes, such as quality of life and emotional well-being, when compared to their siblings or peers.^8^

Among the challenges of caring for AYA patients is the fact that they have unique developmental and psychosocial needs that are distinct from those of pediatric and adult patients^9^ and that may be unrecognized or unmet during their cancer experience.^10^ AYA development is characterized by the establishment of individual identity (eg, sense of self, sexual identity), independence (eg, employment, autonomy from parents, decision making about the future), and peer relationships (eg, intimate relationships, social support networks).^9,11–13^ The impact of cancer on each of these milestones may be profound; patients may regress, delay, or even miss critical developmental steps, leading to long-term negative consequences.^14^

### Defining AYA

Further complicating the clinical care of these patients is the variable definition of AYA and its application to clinical care. The term usually refers to those patients aged 15–39 years at the time of their initial cancer diagnosis, and it is based primarily on the distinct biology of AYA types of cancers;^12^ however, the literature includes age ranges that are inconsistent and that depend on individual malignancies or international perspectives.^15^ The continuum of AYA development is vastly different across its age span. For example, a 17-year-old high school student may have yet to establish her full independence and may struggle with school disruptions, social isolation, and body image, whereas a 30-year-old young mother may focus more on career, family, and financial burdens. Finally, because AYA patients span the gap between pediatric and adult medical centers, their care may be discontinuous or incomplete.^16^

### Defining palliative care

“Palliative care” refers to the total care of a child and his or her family, regardless of disease status.^17^ It aims to improve and support patients’ quality of life by controlling symptoms and alleviating physical, social, psychological, and spiritual suffering.^18^ It involves not only the patient, but also his or her family members, caregivers, friends, significant others, and other social supports.^19^ Whereas the term initially referred to patients at the end of life, its current purview is more holistic and supportive of patients at any stage of a life-threatening illness.^20^ Because a diagnosis of cancer inherently carries with it uncertainty, as well as physical and psychosocial suffering, comprehensive palliative care may be included from the time of diagnosis onward. End-of-life care is simply a component of overall palliative care, when the focus is almost entirely on patient comfort. In this sense, interdisciplinary palliative expertise, either within the primary oncology team or through consultation from a subspecialty palliative-care service, may enhance the quality of care for AYA oncology patients.

Nevertheless, there have been few descriptions or studies of palliative care in the AYA oncology setting.^18,20–24^ As with general AYA oncology care, palliative care for AYA oncology patients is challenged by the diversity of patient needs, ages, and treatment settings. The average age in adult palliative-care units is 60–65 years;^25^ therefore, palliative-care teams in adult centers may have considerably less experience with younger AYA patients. Likewise, only 15.5% of pediatric palliative-care consultations are for patients older than 19 years,^26^ and death in pediatric cancer is relatively uncommon.^1^ Pediatric palliative-care teams may therefore be less comfortable with young adult patients. Furthermore, while some of the physical symptoms and suffering of AYA oncology patients may be similar regardless of age, how clinicians perceive and treat pain may be different in older than in younger patients. Finally, their psychosocial needs with respect to their development, significant others, sexuality, career, and early parenting demands may be vastly different.^20^

The objectives of this review were to describe the scope, challenges, and patient perspectives of palliative care for AYA oncology patients. We suggest directions for future clinical care and research endeavors, with the ultimate goal of providing comprehensive, whole-patient, family-centered care.

## The scope of palliative care in AYAs

Because “palliative care” was viewed as being synonymous with “end-of-life care,” subspecialty palliative-care teams historically were involved only in challenging or “end-stage” cases. However, as the concept of concurrent palliative care has evolved, so too has its scope. While primary oncology teams may be adept at providing primary palliative care, and while patients continue to rely on them for guidance, current models of palliative care suggest that interdisciplinary approaches that include both the primary oncology team and specialized palliative-care staff enable optimal patient and family care, regardless of outcome.^27^

Palliative care in current pediatric and medical oncology settings almost always involves an interdisciplinary approach that includes physicians, nurses, psychosocial clinicians, and others;^28,29^ however, the scope of palliative-care services specifically for AYA oncology patients has not been well defined. This, another AYA-gap, is particularly important, given the special psychosocial needs of AYA patients.

As part of a larger workshop on AYA oncology, a group of Canadian stakeholders, including cancer survivors, caregivers of terminally ill AYA patients, and interdisciplinary health care providers, came together to identify key issues and priorities related to palliative care for AYA patients.^20^ The suggested guidelines were then replicated by the US National Comprehensive Cancer Network AYA Oncology panel.^12^ The reports only briefly described the role of palliative care in the management of pain and physical symptoms; however, this elemental aspect of palliative care has been well described elsewhere.^18,28,29^ Rather, four central themes were recognized by both groups as critical elements of palliative care for AYA oncology patients: (1) psychosocial needs of the patient, family, friends, and caregivers; (2) introduction of palliative care; (3) requisite resources; and (4) advocacy (Table 1). Together, these elements create a strong rationale for an interdisciplinary and integrated approach to palliative care in AYAs.

### Psychosocial needs

AYA oncology patients have unique psychosocial needs. Not only must they grapple with normal developmental milestones relating to identity, body image, sexuality, professional and personal goals, relationships with others, and possibly parenthood, they also must adapt to a serious and life-altering medical illness. AYA cancer survivors report significantly greater psychological distress and fewer positive health beliefs than do younger pediatric or older adult survivors.^8^ They have ongoing physical, social, and emotional challenges, such as physical impairment, infertility, uncertainty, fears about recurrence, interruption of life plans, and discrimination in discrimination in the workplace and in finding insurance.^30,31^ Approximately 25% of AYA survivors manifest post-traumatic stress disorder, and up to 90% have isolated symptoms of post-traumatic stress.^32^

Proposed guidelines for comprehensive and interdisciplinary care of AYAs should also incorporate palliative care. Palliative-care teams are especially equipped to assist patients and families with identifying goals of care,^33–37^ a process that may be critical to AYA patients whose treatment decisions may profoundly impact their long-term psychosocial outcomes and medical morbidity. Furthermore, palliative-care teams may provide continuity during survivorship phases, when patients continue to struggle with the ramifications of their illness. For those patients who die from their cancer, palliative-care teams provide ongoing bereavement care and support to surviving family members.

A key aspect of the care of AYA oncology patients is the recognition of each patient’s level of independence and maturity. Physical and psychosocial regression may be inevitable, and AYA cancer patients may struggle to balance their prior independence with the new confines of their cancer.^18^ In addition, how AYAs make decisions is unique. Often, those under 18 years of age must legally defer to their parents or guardians for medical decision making, and those guardians, as well as the medical staff, may withhold information in an effort to protect the patient.^38^ AYA patients, however, prefer to be informed and involved in shared decision making.^21,39,40^ Allowing them to do so helps maintain their autonomy in an uncontrollable situation.^41^ Palliative-care teams provide expertise in collaborative communication, information sharing, and joint decision making.^33,34,37^ Additionally, AYA-appropriate decision-making tools (eg, the “Voicing My Choices” advanced-care planning guide) have been adapted from pediatric and adult-styled documents to meet the specific needs of AYAs.^35^ These resources may provide additional aids for AYA patients, especially those mature 15- to 18-year-olds who contribute to and direct medical decisions while still legally bound to their parents.

AYAs also may be more willing or capable of accepting and understanding life-limiting decision options than are their healthy peers.^36,39^ The single most important factor that AYA patients cite when making end-of-life decisions is their relationship with others.^36^ They want to be able to direct (or decline) their medical treatment, and to define their care plans, the information their friends and family receive, and how they will ultimately be remembered.^35^ Indeed, involving AYAs in end-of-life decision making may provide the patients with a greater sense of purpose^35^ while alleviating parents’ sense of responsibility and guilt.^37^

Palliative care considers and encourages those relationships and communication between patients and their family, peers, and health care professionals. For example, misunderstandings and discord in end-of-life settings can cause profound patient and caregiver distress;^42^ but advanced-care planning can alleviate caregiver burden^43^ and enable surrogate decision making.^44^

AYA patients may have a variety of support structures, depending on their developmental age. For example, some AYAs may become re-reliant on their parents, whereas others rely on spouses or other AYA patients with cancer. The latter group may experience renewed isolation as curative outcomes become less likely.^20^ This isolation may be mitigated by a physical space in the hospital or clinic that is conducive to social interaction. Both standard AYA oncology practice and palliative-care approaches should integrate such “safe spaces” when possible.^12,20,45^ Alternatively, web-based programs and social networking sites may minimize the feeling of social isolation.^20,46^

Caregivers, family, and friends of AYA oncology patients have similar psychosocial needs. Parents of children with cancer have high levels of psychological distress^47^ and post-traumatic stress disorder,^32^ and these phenomena are no less true for parents of older children. Indeed, parents and friends not only fear the potential loss of the patient’s life, but also grieve the loss of expected milestones. This anticipatory grief begins at the time of diagnosis; concurrent palliative care can alleviate this emotional distress through regular support.^48^ Finally, pediatric and medical oncologists may be challenged by their personal perspectives and discomforts involved with end-of-life communication.^49^ Trained palliative-care providers may be able to provide additional professional support.^50^

### Introduction of palliative care

If they are involved early in the cancer experience, palliative-care teams may assist patients with their initial adjustment to cancer, and then to the challenges that develop over time. Indeed, concurrent palliative care should be introduced early, including during treatment that is intended to cure.^20^ Historically, however, concepts of palliative care have been introduced later, when the cancer is progressive or when patients are receiving end-of-life care. Palliative-care teams are uniquely equipped not only to address existential distress, but also to lead discussions about patient and family wishes, including end-of-life concerns such as place of death.^18^

An additional, and somewhat unique situation in AYA oncology, is when AYA patients are offered a Phase I clinical trial. Opportunities to participate in Phase I research usually arise when curative treatments are lacking; and while these trials are not expected to lead to remission, it is unclear how many AYA patients are fully aware of that implication.^51^ Indeed, while most AYAs who enroll in Phase I research hope that doing so will extend the length of their lives,^52^ 85% of those enrolled in clinical trials also report that helping others is important to them.^53^ Concepts of palliative care should be incorporated into any discussion of Phase I research in order to completely understand and meet the needs and goals of AYA patients.

Unfortunately, the term “palliative care” is associated with negative emotions among caregivers,^54^ and both pediatric and medical oncologists hesitate to make formal referrals to palliative-care teams, because they (1) assume AYA patients are less interested in discussing end-of-life issues^18^ or (2) worry that doing so will decrease hope or increase patient or caregiver distress.^55,56^ There is no evidence that referral to palliative care changes either hope or distress;^22,57^ however, studies have shown that changes in terminology (eg, “supportive care team”) may lead to increased referrals. Furthermore, better definition of palliative care and explanation to families and health care providers about what palliative-care programs offer may improve perceptions about palliative care and increase program utilization.^54^

### Requisite resources

The paucity of evidence-based guidelines for AYA oncology patients has prompted empirical position statements to delineate quality-of-care standards.^16^ These include efficient processes for diagnosis, initiation of treatment, promotion of adherence, and access to health care professionals trained in the biomedical and psychosocial needs of this population. Health care systems have addressed these standards in various age-specific ways;^45^ however, they have been challenged by cultural differences between pediatric and adult centers, as well as by limited resources for meeting the multidisciplinary needs of the AYA population.^58^

These issues extend to the realm of palliative care for AYAs. Patients may be treated in both pediatric and medical oncology settings, and the transition of care is critically important.^18^ In the end-of-life setting, hospice services may be less available to pediatric-aged patients,^59^ but most adult hospice patients are older or seniors, and AYAs may feel additional isolation or distress in such an environment. Balancing available resources with AYA psychosocial needs may be difficult. Furthermore, whereas the majority of AYA oncology patients prefer to die at home,^60^ only 13% actually die there; most die in the hospital.^24^ Those with a hospice that allows concurrent treatment, however, are more likely to die at home.^61^

The financial constraints of end-of-life care may also be prohibitive. Many AYA patients and/or their caregivers must quit or cut back on work during care to meet medical demands. Eighty percent of AYA patients will need assistance with health insurance, disability services or social security,^62^ and many become dependent again on their parents or families to help pay their medical bills.^20^ Palliative-care services for AYAs must therefore be prepared to address these obstacles.

Proposed metrics to demonstrate the use, cost, and efficacy of palliative care for AYA oncology patients include measuring (1) the involvement and timing of palliative-care or hospice teams; (2) the locus of end-of-life care; (3) pain control; (4) caregiver costs, including lost work time and income; (5) care provision and medication costs; (6) quality-of-life measures; and (7) patient and family satisfaction.^63^ Such assessments may ultimately justify the allocation of appropriate resources to this population of patients.

### Advocacy

Health care providers and policy makers may have limited understandings of AYA oncology patents’ needs, in part due to the rare participation of AYAs in clinical trials.^7^ This lack of awareness is compounded by the paucity of palliative-care research conducted among AYA oncology patients. AYA-specific advocacy groups need to be developed to increase awareness and ultimately to improve outcomes.^12,20^

## Overview of the problems associated with the palliative care of AYAs

There are several additional challenges associated with the palliative care of AYAs, in addition to those listed above. First, health care providers may feel that they have insufficient time to have long conversations about end-of-life care.^64^ Among studies conducted in pediatric cancer settings, however, patients and families place more value on the quality of communication than on its quantity or length.^65,66^ Likewise, quality communication in palliative-care settings has been associated with decreased caregiver distress.^67^ While physicians may not feel comfortable predicting a patient’s prognosis,^55^ they do tend to recognize the end-of-life period approximately 100 days before families do.^68^ Families not only report wanting as much prognostic information as possible, but 85% also state that numeric information is helpful; and over one-third state they would want more information than they received.^69^ Finally, families that better understand prognosis are more likely to report high quality of care.^68^

Parents and caregivers may seek guidance in talking to their loved ones about death. No parents who talk to their dying adolescents about death report regret at having done so, while 27% of those who avoid the topic wish they had not.^42^ Furthermore, parents are 63% less likely to be distressed if their AYA oncology patient is in the room during difficult conversations.^69^ This finding may be due to the fact that having the patient in the room opens the lines of communication and enables transparent discussions of goals and priorities. Current evidence suggests that the timing of end-of-life discussions among AYA oncology patients often occurs too close to death to allow patients to psychologically prepare.^22^

Most AYA oncology patients ultimately die of disease progression; however, half of those who die in the hospital die in the intensive care unit, where they are more likely to have treatment-related causes of death.^22^ During the last week of life, patients experience an average of four physical symptoms. Depending on their type of cancer, these most often include pain and shortness of breath.^24^ To our knowledge, the prevalence of these symptoms is similar in AYAs and younger pediatric patients;^70^ however, appropriately controlling older patients’ complaints may be more challenging. AYAs, for example, may be more likely to be stigmatized as “drug-seeking,” and providers may be less comfortable prescribing high, but weight-appropriate, levels of opiates.

At the end of life, almost all patients experience psychological symptoms such as sadness, anxiety, fear (of being alone, of death, or of pain), and guilt.^24^ AYAs have significantly higher rates of anxiety and depression than do younger age groups.^22,70^ Palliative care must therefore include comprehensive relief of physical and emotional suffering, including nutrition, hydration, sedation, treatment cessation, and place of death.^18^ Investigations have shown that advanced-care planning documents are helpful for terminally ill AYA patients with cancer;^35,71^ however, the use of such documents requires, first, providers willing to create them, and second, the resources required to implement them.^18^

Perhaps the most difficult and poignant challenge is what to do when AYA patients and their caregivers, family members, or health care professionals disagree. This ethical dilemma has been described for adolescent, mature minors,^72^ but navigating such disagreements remains daunting. Palliative-care teams often include bioethical perspectives that may assist in these situations.^18^

Finally, AYA oncology patients are willing to participate in clinical trials regarding palliative or end-of-life care.^21^ They understand that such endeavors will ultimately help improve not only their own quality of life,^22,52,73^ but also that of their family and other AYAs who may be affected by cancer in the years to come.^36,52,53^ Researchers must strive to conduct rigorous studies to ultimately improve the palliative-care experience and standard of care for AYAs with cancer.

## Patient perspectives

Adolescents over 14 years of age cognitively understand that death is permanent and irreversible, and AYAs with cancer may have had personal experience with the deaths of fellow cancer patients. However, AYA patient perspectives about palliative and end-of-life care vary; exploring their individual preferences may improve care.^12^ Almost all patients state that they “think about their relationship to others” when they make end-of-life decisions, and the majority wish to avoid adverse events and/or stop their cancer treatment.^36^ AYAs also use a variety of coping mechanisms, most commonly involving supportive coping.^74^ Indeed, AYAs who report higher levels of social support have superior emotional health and existential quality of life, and less severe grief.^75^

In a retrospective study of decision making among cancer survivors (ages 11–18 years at the time of their diagnosis), 96% of participants agreed that adolescent minors should have the right to be informed of the terminality of their condition; however, only 86% said they would want to know. Compared to a separate cohort of previously healthy AYAs, those with histories of cancer were more likely to endorse nontreatment decisions or to say that physician-assisted suicide was okay.^39^

## Future directions

Evidence-based guidelines for palliative care in AYAs with cancer are limited and in need of development. The National Institutes of Health, the Institute of Medicine, and the National Palliative Care Research Center have all cited AYA palliative care as a research priority, in order to better understand the emotional, spiritual, and practical needs of AYA oncology patients and their caregivers. Future endeavors must not only describe patient perspectives and patient-centered outcomes, but also how to develop interventions to foster more functional outcomes during and after cancer.

Meanwhile, clinical standards must be developed for the palliative care of AYA oncology patients. These must include comprehensive care programs that involve interdisciplinary teams to meet patient and caregiver psychosocial needs. Standards must be developed that not only cross between pediatric and medical oncology centers, but also incorporate the care and support of both caregivers and health care professionals.

## Discussion

AYA oncology and palliative care both face challenges in their definitions, clinical applications, and research endeavors. Investigations of the two combined are few, but they provide a starting point for establishing standards of clinical care and future investigations. First, the scope of palliative care for AYA oncology patients must not only include exceptional pain and symptom management, but also address patient and caregiver psychosocial needs. The concept of palliative care and its partnership with primary oncology teams must be introduced early and appropriately. AYA oncology programs must develop and retain the resources to involve an interdisciplinary, palliative-care team; such accomplishments might only be attained through advocacy efforts that increase patient and health care provider awareness of palliative care, its role, and how it may support whole-patient care.

Second, while palliative care may be considered optional for some well AYA oncology patients, it is imperative for those with advanced illness. Among AYAs in the USA, cancer remains the leading cause of death,^5^ and quality end-of-life care must be a priority. Patients may hesitate to talk openly about death, regardless of their prognosis, but most understand that “cancer” connotes a risk of dying. Palliative-care teams may facilitate discussion of patient and caregiver fears, preferences, and wishes. Indeed, AYAs with serious medical conditions other than cancer have suggested that advanced-care planning informs patient options and enables appropriate goal-setting.^37^ These outcomes may lead, in turn, to improved coping and adjustment for both patients and caregivers.

AYA patients are willing to engage in palliative-care research. Future endeavors must not only expand on the limited patient-reported perspectives, they must also think creatively about innovative ways to provide palliative care and meet the needs of AYAs with cancer. For example, social media may be critical, but under-studied sources of support for this population. Likewise, how AYAs cope throughout their cancer experience, how their needs change over time, and what will promote more functional outcomes in this somewhat vulnerable group are all unclear.

Palliative care for AYAs with cancer has the potential to meet a wide variety of patient needs, from pain and symptom management to psychosocial support, supportive communication, goal setting, and advanced-care planning. The benefits of palliative-care involvement are far-reaching: not only do these teams alleviate immediate physical and psychosocial suffering, but they also may have long-term impacts on patients and their communities, ultimately enabling better coping and adjustment to life after cancer.

This review provides a broad impression of the existing literature describing or investigating palliative care in AYA oncology patients. Put together, the evidence suggests that palliative care is not only needed, but can also be critically beneficial to patients, families, and health care professionals. As we increase public and professional awareness of the needs and applications of palliative care for AYA patients with cancer, we will ultimately enable better psychosocial outcomes for the AYA patients and their larger communities.

## Figures and Tables

**Table 1:** Key issues and priorities of palliative care for adolescents and young adults with cancer^a^

**Patient** Psychosocial needs Tailor approach to patient’s level of independence and maturity Facilitate social support, peer relationships, and interactions Minimize isolation with physical or web-based “space” Provide age-appropriate, multidisciplinary supportive care Family and friends Provide regular, interdisciplinary support Recognize and support anticipatory grief, if applicable Professional caregivers Provide support and debriefing to maintain work life balance and wellness and avoid burnout **Introduction of palliative care** Provide early, regular, and routine screening for psychosocial needs **Requisite resources** Should address financial needs and constraints Facilitate patient preferences, including location of death Involve interdisciplinary approach, across institutions if applicable **Advocacy** Increase awareness of the special needs of AYA oncology patients among health care professionals and those responsible for policy

a Adapted from recommendations based on the international consensus group meeting.^2,20^
